# Cutaneous complications of molecular targeted therapy used in oncology

**Published:** 2016

**Authors:** I Lupu, N Voiculescu, N Bacalbasa, I Cojocaru, V Vrancian, C Giurcaneanu

**Affiliations:** * Department of Dermatology, “Elias” University Hospital, Bucharest, Romania; ** “Carol Davila” University of Medicine and Pharmacy, Bucharest, Romania; *** Bucharest University Emergency Hospital, Bucharest, Romania

**Keywords:** cutaneous adverse reactions, molecular targeted therapy, inhibitors of EGF receptors, oncology

## Abstract

The new molecular targeted therapy has been developed over the past decades by using the molecular targeted molecular changes discovered in specific types of cancer. Unfortunately, most of these agents (epidermal growth factor receptors, multi-targeted small molecule tyrosine kinase inhibitors, monoclonal antibodies) have severe cutaneous adverse reactions, that not only interfere with the patient’s quality of life, but also are dose–limiting and may require treatment interruptions. These cutaneous complications and their management must be very well known by any oncologist and dermatologist who treat oncologic patients.

Abbreviations: EGFR = epidermal growth factor receptors, EGFRI = epidermal growth factor receptors inhibitors

## Introduction

 Oncological therapies have numerous side effects, both systemic and cutaneous. Patients treated for cancer, either with classic chemotherapeutic agents or with novel targeted antineoplastic therapies, have a high risk of developing adverse reactions involving the skin, mucous membranes, hair, and nails [**1**,**2**,**3**]. The correct diagnosis of a cutaneous adverse reaction to a certain oncologic drug requires a thorough differential diagnosis with cutaneous reactions to other drugs used by the patient, dermatological diseases unrelated to the oncological therapy, cutaneous metastasis, paraneoplastic signs or graft versus host disease (if a transplant was performed) [**1**].

Cutaneous adverse reactions to oncological therapy impair the patients’ quality of life, emotional well-being and sometimes can be so severe that require dose reduction, temporary or permanent interruption of the treatment. The oncologist and the dermatologist treating the oncologic patients must know how to recognize and treat these adverse reactions, in order to increase the patient’s well-being and improve his adherence to therapy.

The classical chemotherapeutic agents have been used for the past six decades and their cutaneous adverse reactions are well known. They include infusion reaction [**4**], diffuse or localized pigmentary changes of the skin, nails, and mucous membranes [**5**], nail disorders (Beau’s lines, pigmentary changes, onycholysis, paronychia) [**2**], alopecia, photosensitivity [**6**], stomatitis, radiation recall dermatitis or radiation enhancement [**7**], hand-foot syndrome [**8**], subacute cutaneous lupus erythematosus [**9**] and scleroderma-like changes [**10**], neutrophilic eccrine hidradenitis [**11**], morbilliform rashes [**12**], fixed drug eruptions, exfoliative dermatitis, erythema multiforme, Steven Johnson syndrome toxic epidermal necrolysis [**14**]. Very rare cutaneous reactions to certain chemotherapeutic agents are leg ulcers to hydroxyurea [**15**], Raynaud’s phenomenon, dermatomyositis like-reaction, paraneoplastic pemphigus-like phenomena to fludarabine, lichenoid eruption to hydroxyurea, eosinophilic cellulitis to cladribine, porphyria, inflammation of benign lesions, or reactivation of varicella-zoster virus [**1**,**2**].

Novel antineoplastic therapy strategies have been developed in the past two decades after detecting molecular changes in certain types of cancer. These molecularly targeted agents are associated with new specific cutaneous reactions, sometimes so severe that may require reducing the doses or stopping the therapy altogether [**16**].

These biologic and molecularly targeted antineoplastic agents can be summarized in four main classes: epidermal growth factor receptor inhibitors, small molecule kinase signal transduction inhibitors, monoclonal antibodies that target molecules other than EGFR and cytokine agents (colony stimulating factors, interferons, and interleukin-2). This article describes the cutaneous toxicities associated with some of these agents, which are more frequently used in therapy. Fortunately, the presence and the severity of some of these dermatological side effects seem to have a positive correlation with response to treatment and overall survival, especially for EGFR inhibitors [**18**].

### Epidermal Growth Factor Receptor Inhibitors

The epidermal growth factor receptor inhibitors are targeted chemotherapeutic agents approved for the treatment of advance stage epithelial cancers like non-small cell lung cancer, colorectal cancer, breast cancer, pancreatic cancer and head and neck squamous cell carcinoma. They include two subclasses: monoclonal antibodies given intravenously (cetuximab, panitumumab) that target the extracellular tyrosine kinase domain of EGFR and small molecule tyrosine kinase inhibitors (gefitinib, erlotinib, lapatinib, and afatinib) that are orally administered and target the intracellular domain [**17**]. Because EGFR is expressed in the skin and adnexal structures, EGFR inhibitors are associated with significant cutaneous adverse reactions, mainly acneiform eruption and xerosis, but also paronychia, abnormal scalp, facial hair, and eyelash growth, maculopapular rash, mucositis and post inflammatory hyperpigmentation [**19**]. The common cutaneous reactions to EGFR inhibitors are labeled as the PRIDE syndrome (papulopustules and/ or paronychia, regulatory abnormalities of hair growth, itching, dryness due to the epidermal growth factor receptor inhibitors) [**27**].

### Acneiform eruption

The acneiform eruption is the most common cutaneous reaction seen in patients receiving EGFR inhibitors, occurring in 24-62% or patients taking gefitinib, 49-67% of those on erlotinib, 75-91% of the patients taking cetuximab. Only 5-10% of the patients receiving EGFR inhibitors develop severe reactions [**16**,**20**,**21**]. Despite its name, this rash differs from acne from both the clinical and histopathological point of view. It manifests itself as follicular centered erythematous papules and pustules, without comedones, that predominately affect the seborrheic areas (face, scalp, upper trunk, the “V” region of the chest and neck), the lower trunk, extremities, and buttocks, sparing the periorbital region, palms and soles [**22**]. Unlike acne, lesions can be associated with pruritus (significant in one-third of the patients), pain, stinging, and irritation [**22**,**23**,**24**]. 

The acneiform eruption is dose-dependent, and the onset typically occurs within the first two weeks of treatment [**19**,**24**]. The rash evolves through four stages: dysesthesia accompanied by erythema and edema in the first week; papulopustular eruptions in the second and third week, crusting during weeks 3 and 4 and persistent erythema, xerosis and telangiectasia after more than a month [**28**]. Most patients see a complete/ significant resolution of lesions despite continuing treatment with EGFR inhibitors. The lesions completely disappear one month after treatment discontinuation [**24**,**25**]. Long-term cutaneous sequelae include post-inflammatory hyperpigmentation, telangiectasia, and erythema [**24**]. Unfortunately, some patients develop persistent severe acneiform reactions that, unless properly managed, require dose adjustment, interruption, or discontinuation. Some studies show a consistent positive correlation between the severity of the acneiform rash and the response to treatment [**14**,**15**]. 

The initial papulopustular lesion was considered a sterile neutrophilic suppurative infiltrate, Staphylococcus aureus colonizing the late phases of the eruption [**20**]. The treatment protocols include topical and systemic antibiotic therapy, without testing the pustules for bacteria or fungus. Cultures swabs from pustules with antibiograms are performed only in cases refractory to treatment.

In a pilot study, taking place in the Oncology Departments of the University Emergency Hospital of Bucharest and the Romanian National Oncology Institute, 43 patients that developed acneiform rashes while receiving EGFR inhibitors were enrolled. These papulopustular reactions were classified into early (33 cases - 25 patients taking cetuximab, 6 erlotinib and 2 with lapatinib), when the eruption occurred 4 to 14 days after initiating the therapy (mean 7 days), and late (10 cases - seven patients receiving cetuximab and 3 erlotinib), when it occurred more than 150 days after the start of therapy. Methicillin sensitive Staphylococcus aureus was detected in 27 of 33 patients with the early onset of papulopustular folliculitis (81.81%). Two of the patients developed Enterobacter in cultures, 1 patient Citrobacter diversus and 1 patient Acinetobacter. The antibiogram revealed Staphylococcus resistant to tetracyclines both in pustules (63.63% of the cases) and in the patient’s nasal cavity (60% of the cases). In the late onset group, all the cultures were positive for methicillin-sensitive Staphylococcus aureus. The cultures were positive for Staphylococcus nose in 5 of the 11 patients (45.45%). Tetracycline resistant Staphylococcus was isolated in both the pustules (62.96% of cases) and the nasal cavity (64.7% cases) of these patients.

### Paronychia

Paronychia (painful inflammation of the nail fold) and periungual pyogenic-like lesions are the frequent adverse reaction to EGFR inhibitors, appearing after one or two months of treatment in 10-15% of the patients [**27**] due to the direct inhibition of keratinocytes in the nail matrix [**19**]. Nail matrix inflammation can also cause nail discoloration, pitting, ingrowth of nails, and onycholysis [**19**]. The fist digit is affected the most [**19**]. Paronychia is not considered an infectious process, but secondary infection with Staphylococcus aureus or coagulase-negative, Gram-positive bacteria (nosocomial colonization) can occur [**16**]. Culture swabs are recommended to determine the proper antibiotic treatment. 

### Xerosis and fissure

Xerosis is the second most common adverse reaction, affecting up to 35% of the patients receiving EGFRI [**31**] and is caused by the abnormal keratinocyte differentiation, which induces an altered stratum corneum, a decrease in moisture retention and a reduction in epidermal loricrin [**16**]. Xerosis usually limits itself to areas affected by papulopustular rash, but severe cases like asteatotic eczema and acral fissures are not uncommon [**32**]. Xerosis of vaginal and perineal mucosa has been reported [**28**].

### Regulatory hair changese

A variety of hair changes have been described usually after 2-5 months of therapy with EGFRI. They include trichomegaly (elongation and curling of eyelashes) that may cause corneal irritation and ulceration, marked increase in the length of the eyebrows, hair abnormalities (scalp and extremity hair becoming brittle, finer, and curly), scarring/ non-scarring alopecia, hypertrichosis, facial hirsutism [**19**,**27**,**30**]. Most of these hair changes are temporary. Hair resumes its normal growth usually 1 month after ceasing therapy [**27**].

### Other cutaneous reactions

Pruritus can affect more than 50% of the patients, creating a great discomfort.

Pruritic maculopapular rash appears later than the papulopustular eruption and mainly affects the face and limbs. It is accompanied by a dry pulpitis of the fingers, feet xerosis, and photosensitivity [**31**]. 

Anaphylactic reactions have been reported in up to 3.5% of patients receiving therapy with cetuximab and 1% of the patients taking panitumumab [**27**]. 

Other cutaneous adverse reactions to EGFRI include aphthous-like ulcerations of the oral and nasal mucosa, moderate mucositis, stomatitis, and photosensitivity [**20**,**33**]. Rare cases of bullous and exfoliative eruptions, Steven-Johnson syndrome or toxic epidermal necrolysis [**35**,**39**], ocular complications (dry eye, corneal abrasions) [**34**], small vessel vasculitis, purpura on the lower extremities [**35**,**39**], necrolytic migratory erythema-like rash [**37**], transient acantholytic dermatosis [**38**] were reported. 

Despite the other theories, patients receiving both cetuximab and radiation therapy do not show an increased risk of radiation dermatitis or mucositis [**40**].

### Small molecule kinase signal transduction inhibitors

Small molecule kinase signal transduction inhibitors inhibit tyrosinkinase in a series of ways: 

**1.Imatinib**, **dasatinib**, **ponatinib**, **nilotinib**, **and bosutinib** inhibit signal transduction through the BCR-ABL fusion protein, c-KIT, the platelet-derived growth factor (PDGFR) family of TKs, and the SRC family of TKs. 

**Imatinib**was the first drug developed to inhibit the BCR-ABL tyrosine kinases in Philadelphia-chromosome-positive chronic myelogenous leukemia, c-kit in rare gastrointestinal stromal tumors (that present KIT mutations) and several platelet-derived growth factor receptors (PDGFRs) in other forms of cancer [**41**]. Superficial edema is characteristic to imatinib, mainly affecting the periorbital area (60% of the cases, causing epiphora, chemosis, and conjunctivochalasis) and the extremities. In rare cases, edema can affect the central part of the body [**27**,**41**]. The most common cutaneous adverse reaction to imatinib is a dose dependent maculopapular rash that affects the torso, forearms, rarely the face [**42**,**43**]. Low-grade rashes resolve themselves spontaneously while continuing therapy, but severe skin reactions may require a temporary discontinuation of the treatment followed by the reintroduction, at a lower daily dose in association with oral corticosteroids [**44**]. High doses of imatinib may cause extremely severe reactions, including the Steven-Johnson syndrome that requires a permanent drug discontinuation [**44**,**45**]. Patients receiving imatinib may develop localized, spotted, or diffuse pigmentary changes of the skin, hair, nails, and oral mucosa [**46**,**47**]. Hypopigmentation has been observed in up to 33% of the patients treated with imatinib [**48**], whereas hyperpigmentation only in 3.6% of the cases [**43**]. Hypopigmentation is caused by the inhibition of the c-kit, that regulates the melanocytes development, migration, and survival [**41**], being reversible with drug reduction or discontinuation, whereas hyperpigmentation is caused by the deposition of drug metabolites containing melanin and iron [**49**]. Other reactions include urticarial, lichenoid, pityriasiform, and psoriasiform rashes [**41**], exacerbations of the existing psoriasis [**54**], acute and generalized exanthematous pustulosis (AGEP) [**50**], Sweet syndrome (acute febrile neutrophilic dermatosis) [**51**], neutrophilic eccrine hidradenitis, and neutrophilic panniculitis, mycosis fungoides like reaction, follicular mucinosis, malpighian epithelium, porphyria cutanea tarda, and pseudoporphyria [**41**,**52**], photosensitization [**53**]. In rare cases, patients may develop small vessel vasculitis, erythema nodosum, and a graft-versus-host-like skin reaction [**55**]. 

**Dasatinib,nilotinib,ponatinib**and**bosutinib**are second-generation multi-targeted TK inhibitors that are used for treatment of Ph+CML with acquired BCR-ABL mutations [**41**]. Cutaneous adverse reactions appear in 35% of the patients treated with dasatinib [**55**] and include localized and generalized erythema, papular eruptions, exfoliative rash, pruritus, hyperhidrosis, alopecia, xerosis, acne, urticaria, dermatitis, photosensitivity, nail disorders and pigmentary changes, skin ulcers, palmoplantar erythrodysesthesia syndrome. Rare case of panniculitis, acute febrile neutrophilic dermatosis, and bullous disorders were reported [**27**,**41**]. 30% of the patients treated with dasatinib develop pleural effusions [**27**,**41**]. Patients treated with nilotinib, develop a maculopapular rash in 28% of the cases, pruritus in 24%, and xerosis alopecia, and bullous Sweet syndrome in 12% of the cases [**41**,**56**]. Ponatinib has been associated with rash and dry skin in up to 40% of the patients [**57**]. Bosutinib causes adverse skin reactions in 20-44% of the patients, including erythema, maculopapular eruption, pruritic rash, allergic dermatitis, acne, folliculitis, and skin exfoliation [**58**].

**2.Sorafenib**and**sunitinib**are multikinase inhibitors that specifically target tumor cell angiogenesis and proliferation, by inhibiting PDGFR, vascular endothelial growth factor receptor (VEGFR) and KIT. Sorafenib also targets Raf kinase [**59**]. Sorafenib is used in the therapy of renal cell carcinoma, colon, hepatic or pancreatic cancer, non-small cell lung cancer, while sunitinib was approved for renal cell carcinoma, gastrointestinal tumor, colon cancer and breast cancer [**27**]. 74% of the patients receiving sorafenib and 81% of those taking sunitinib develop cutaneous adverse reactions, due to either the results of vessel damage by inhibition of PDGFR and VEGFR, or the drug extravasation into the skin and mucosae [**59**]. 

**Hand foot syndrome**(HSFR) is the most common cutaneous adverse effect seen in patients treated with sorafenib (62% of the cases) and sunitinib (28%), especially at higher doses [**60**]. While acral erythema associated with conventional cytotoxic chemotherapy is characterized by symmetric, well demarcated edema and erythema of the palms and soles that can blister and ulcerate, hand-foot skin reactions seen in patients treated with agents targeting VEGFR, manifest with localized painful blister or hyperkeratosis patches in areas of friction or repetitive trauma (heel, lateral aspects of the soles, lateral sides of the fingers and the periungual regions, web spaces, dorsal surfaces of the hands and feet), that start within the first two to four weeks of therapy. Hyperkeratosis can the only manifestation of the hand-foot syndrome [**61**]. Biopsy specimens show epidermal acanthosis, parakeratosis and dyskeratosis with band like areas of necrotic keratinocytes and a superficial perivascular lymphocytic infiltrate in the dermis [**61**]. Both the acral erythema and HFSR can be accompanied by paresthesia, tingling, burning, painful sensations on the palms and soles.

**Stomatitis**is the second most common cutaneous reaction from treatment with sorafenib (26%) and sunitinib (36%). Stomatitis appears early in the course of treatment and is directly correlated to the severity of HFSR [**62**].

**Hair changes** 26% of the patients taking sorafenib and 6% of those taking sunitinib develop alopecia (reversible if treatment is discontinued) 2 to 28 weeks after the onset of therapy [**62**]. Sorafenib causes hair to become fragile, curly and pigmented, while sunitinib causes hair depigmentation, completely reversible one month after treatment discontinuation. The patients’ hair receiving intermittent therapy may show alternative bands of hyperpigmentation and depigmentation [**62**].

**Cutaneous squamoproliferative lesions.** Sorafenib has been associated with the development of new squamous cell carcinomas, keratoacanthomas, and inflammation of preexisting actinic keratosis in 10% of the patients [**71**,**72**]. Therefore, a close follow-up is mandatory. Although cases of spontaneous regression of keratoacanthomas after drug discontinuation were reported [**73**,**74**], if a suspicious lesion is identified it should be treated as in the case of a patient who does not receive oncologic therapy, usually with a complete excision.

**Other cutaneous adverse effects.** Seborrheic dermatitis-like facial and scalp erythema were reported early in the course of treatment with sorafenib (63%) and sunitinib. It disappears 2 months after the treatment discontinuation [**63**]. 24% of the patients treated with sunitinib develop facial edema [**63**]. More than 50% of the patients treated develop transient scalp dysesthesia [**64**]. Yellow skin pigmentation of the face (28% of the patients taking sunitinib) developed usually after a month of therapy is due to the yellow color of sunitinib [**27**]. It resolves itself two months after ceasing treatment.

Other cutaneous adverse effects seen with sorafenib and sunitinib include subungual splinter hemorrhages, seen in the first months of therapy in up to 70% of the patients receiving sorafenib and 25% of those receiving sunitinib [**63**], periungual erythema [**67**,**68**], erythema multiforme and erythema multiforme-like eruptions [**66**], Stevens-Johnson syndrome, eruptive melanocytic nevi and drug-induced lentigines secondary to sorafenib [**62**,**65**], pyoderma gangrenosum [**69**], generalized keratosis pilaris like eruption, epidermal cysts, nipple hyperkeratosis and/ or dysesthesia [**62**,**63**,**64**] or dyskeratotic plaque with milia [**70**].

**3. Gene therapy**

Vemurafenib and dabrafenib are potent inhibitors of the kinase domain in mutant BRAF, approved for the treatment of metastatic melanoma with a V600E BRAF mutation. Cutaneous reactions similar to the adverse events caused by EGFR inhibitors (RAF mediates EGFR signaling pathways) are present in 74% of the patients [**50**] and include dose dependent papulopustular rash (18% of the patients taking vemurafenib, 27% of those dabrafenib) [**81**,**82**], photosensitivity (12% of the patients taking vemurafenib) [**82**], xerosis, pruritus, paronychia [**80**], alopecia and hair follicle alterations, hyperkeratosis [**77**], pyogenic granulomas [**24**,**29**]. Patients receiving vemurafenib and dabrafenib may develop verrucous keratosis, acantholytic dermatosis, seborrheic keratosis, verruca vulgaris, and hypertrophic actinic keratosis, but also cutaneous squamous cell carcinoma and/ or keratoacanthoma (18-26%) 2 to 14 weeks into treatment [**26**].

**Trametinib** is an inhibitor of the mitogen activated protein kinase (MAPK) MEK1 and MEK2 enzymes approved for the treatment of unresectable melanoma with a BRAF V600E or V600K mutation [**80**], whose cutaneous adverse effects include acneiform rash (75% of the cases), pruritus (27%), and xerosis (22%) [**13**]. A decreased incidence of squamous cell carcinoma side effects was observed with this drug [**80**].

### Monoclonal antibodies

Monoclonal antibodies targeting other molecules than EGFR, like **rituximab, alemtuzumab, bevacizumab, trastuzumab, ofatumumab, obinutuzumab, nivolumab, pertuzumab, or ipilimumab, ** are well-tolerated oncologic therapies, except for allergic reaction. **Infusion reactions** are frequent with rituximab and alemtuzumab and they develop after the first or second exposure to the agent, 30 minutes to 2 hours prior infusion [**74**]. Rituximab may cause **serum sickness** one or two weeks after treatment initiation that manifests with fever, arthralgia, and morbilliform rash. Laboratory findings showed depressed C3 and C4 levels, elevated erythrocyte sedimentation rate, and C-reactive protein. A non-specific erythematous rash has been described in patients receiving any of the monoclonal antibodies mentioned above [**2**].

**Ipilimumab** is an immune-modifying monoclonal antibody that promotes unrestrained T cell activation against tumor cells, used as an intravenous treatment for metastatic melanoma and with promising results in trials on ovarian cancer, prostate cancer, and metastatic renal cancer. Immune related adverse events like dermatitis and pruritus, enterocolitis, hepatitis and less frequently uveitis, iridocyclitis, neuropathy, hypophysitis and Guillaine-Barre syndrome, affect 64% of the patients treated with this drug, but serious cases are seen only in 10% of them [**75**,**76**]. Cutaneous adverse reactions appear after 3 to 4 weeks of therapy and gastrointestinal and hepatic ones after 6 to 7 weeks [**77**]. The reactions are dose dependent and may correlate with the treatment’s efficacy. Rare cases of Stevens-Johnson syndrome, toxic epidermal necrolysis, full thickness dermal ulceration or dermatomyositis have been reported in patients with advanced melanoma treated with ipilimumab [**78**,**79**]. 

## Conclusion

The emergence of new molecularly targeted therapies has brought about the increase of cutaneous adverse effects’ incidence among oncologic patients. The severity of these cutaneous side effects, besides altering the patients’ quality of life and emotional well-being, can require dose-alteration or lead to therapy discontinuation. Due to the life-saving nature of these therapies, it is critical that dermatologists recognize the reactions, understand their mechanisms, and find the appropriate treatment for each case.

### Disclosure

None

### Acknowledgement

This paper is supported by the Sectoral Operational Programme Human Resources Development (SOP HRD), financed from the European Social Fund and by the Romanian Government under the contract number POSDRU/159/1.5/S/137390”.

## References

[1] Reyes-Habito CM, Roh EK (2014). Cutaneous reactions to chemotherapeutic drugs and targeted therapies for cancer: part I. Conventional chemotherapeutic drugs. J Am Acad Dermatol..

[2] Payne AS, James WD, Weiss RB (2006). Dermatologic toxicity of chemotherapeutic agents. Semin Oncol.

[3] Remlinger KA, James WD, Weiss RB (2003). Cutaneous reactions to chemotherapy drugs: the art of consultation. Arch Dermatol..

[4] Lenz HJ (2007). Management and Preparedness for Infusion and Hypersensitivity Reactions. The Oncologist.

[5] Susser WS, Whitaker-Worth DL, Grant-Kels JM (1999). Mucocutaneous reactions to chemotherapy.. J Am Acad Dermatol..

[6] Alley E, Green R, Schuchter L (2002). Cutaneous toxicities of cancer therapy. Curr Opin Oncol.

[7] Phillips TL, Fu KK (1976). Quantification of combined radiation therapy and chemotherapy effects on critical normal tissues.. Cancer.

[8] Miller KK, Gorcey L, McLellan BN (2014). Chemotherapy-induced hand-foot syndrome and nail changes: a review of clinical presentation, etiology, pathogenesis, and management. J Am Acad Dermatol..

[9] Weger W, Kränke B, Gerger A (2008). Occurrence of subacute cutaneous lupus erythematosus after treatment with fluorouracil and capecitabine. J Am Acad Dermatol..

[10] Bessis D, Guillot B, Legouffe E, Guilhou JJ (2004). Gemcitabine-associated scleroderma-like changes of the lower extremities. J Am Acad Dermatol..

[11] Brehler R, Reimann S, Bonsmann G, Metze D (1997). Neutrophilic hidradenitis induced by chemotherapy involves eccrine and apocrine glands.. Am J Dermatopathol..

[12] Heidary N, Naik H, Burgin S (2008). Chemotherapeutic agents and the skin: An update. J Am Acad Dermatol..

[13] Kim KB, Kefford R, Pavlick AC (2013). Phase II study of the MEK1/MEK2 inhibitor Trametinib in patients with metastatic BRAF-mutant cutaneous melanoma previously treated with or without a BRAF inhibitor. J Clin Oncol..

[14] Castaneda CP, Brandenburg NA, Bwire R (2009). Erythema multiforme/Stevens-Johnson syndrome/toxic epidermal necrolysis in lenalidomide-treated patients. J Clin Oncol..

[15] Weinlich G, Schuler G, Greil R (1998). Leg ulcers associated with long-term hydroxyurea therapy. J Am Acad Dermatol..

[16] Reyes-Habito  CM, Roh EK (2014). Cutaneous reactions to chemotherapeutic drugs and targeted therapy for cancer: Part II. Targeted therapy. J Am Acad Dermatol..

[17] Ciardiello F, Tortora G (2008). EGFR antagonists in cancer treatment. The New England. Journal of Medicine.

[18] Liu HB, Wu Y, Lv TF (2013). Skin rash could predict the response to EGFR tyrosine kinase inhibitor and the prognosis for patients with non-small cell lung cancer: a systematic review and meta-analysis. PLoS ONE.

[19] Hu JC, Sadeghi P, Pinter-Brown LC (2007). Cutaneous side effects of epidermal growth factor receptor inhibitors: clinical presentation, pathogenesis, and management. J Am Acad Dermatol..

[20] Busam KJ, Capodieci P, Motzer R (2001). Cutaneous side-effects in cancer patients treated with the antiepidermal growth factor receptor antibody. Br J Dermatol..

[21] Jatoi A, Green EM, Rownland KM Jr. (2009). Clinical predictors of severe cetuximab-induced rash: observations from 933 patients enrolled in north central cancer treatment group study. N0147. Oncology..

[22] Roé E, García Muret MP, Marcuello E (2006). Description and management of cutaneous side effects during cetuximab or erlotinib treatments: a prospective study of 30 patients.. J Am Acad Dermatol..

[23] Joshi SS, Ortiz S, Witherspoon JN (2010). Effects of epidermal growth factor receptor inhibitor-induced dermatologic toxicities on quality of life. Cancer.

[24] Li T, Perez-Soler R (2009). Skin toxicities associated with epidermal growth factor receptor inhibitors. Target Oncol..

[25] Boussemart L, Boivin C, Claveau J (2013). Vemurafenib and radiosensitization. JAMA Dermatol..

[26] Anforth R, Fernandez-Peñas P, Long GV (2013). Cutaneous toxicities of RAF inhibitors. Lancet Oncol..

[27] Heidary N, Naik H, Burgin S (2008). Chemotherapeutic agents and the skin: an update. J Am Acad Dermatol..

[28] Lynch TJ Jr., Kim ES, Eaby B, Garey J, West DP, Lacouture ME (2007). Epidermal growth factor receptor inhibitor-associated cutaneous toxicities: an evolving paradigm in clinical management. Oncologist..

[29] Sammut SJ, Tomson N, Corrie P (2014). Pyogenic granuloma as a cutaneous adverse effect of vemurafenib. N Engl J Med..

[30] Dueland S, Sauer T, Lund-Johansen F (2003). Epidermal growth factor receptor inhibition induces trichomegaly. Acta Oncol..

[31] Balagula Y, Garbe C, Myskowski PL (2011). Clinical presentation and management of dermatological toxicities of epidermal growth factor receptor inhibitors. Int J Dermatol..

[32] Tan EH, Chan A (2009). Evidence-based treatment options for the management of skin toxicities associated with epidermal growth factor receptor inhibitors. Ann Pharmacother.

[33] Luu M, Lai SE, Patel J (2007). Photosensitive rash due to the epidermal growth factor receptor inhibitor erlotinib. Photodermatol Photoimmunol Photomed.

[34] Abdullah SE, Haigentz M Jr., Piperdi B (2012). Dermatologic toxicities from monoclonal antibodies and tyrosine kinase inhibitors against EGFR: pathophysiology and management.. Chemother Res Pract..

[35] Lacouture ME, Anadkat MJ, Bensadoun RJ, Bryce J, Chan A, Epstein JB (2011). Clinical practice guidelines for the prevention and treatment of EGFR inhibitor-associated dermatologic toxicities.. Support Care Cancer..

[36] Lin WL, Lin WC, Yang JY (2008). Fatal toxic epidermal necrolysis associated with cetuximab in a patient with colon cancer. J Clin Oncol..

[37] Trojan A, Jacky E, Follath F, Dummer R (2003). Necrolytic migratory erythema (glucagenoma)-like skin lesions induced by EGF-receptor inhibition.. Swiss Med Wkly.

[38] Tscharner GG, Buhler S, Borner M, Hunziker T (2006). Grover’s disease induced by cetuximab.. Dermatology.

[39] Kurokawa I, Endo K, Hirabayashi M (2005). Purpuric drug eruption possibly due to gefinitib (Iressa). Int J Dermatol..

[40] Bonner JA, Harari PM, Giralt J (2006). Radiotherapy plus cetuximab for squamous-cell carcinoma of the head and neck.. N Engl J Med..

[41] Amitay-Laish I, Stemmer SM, Lacouture ME (2011). Adverse cutaneous reactions secondary to tyrosine kinase inhibitors including imatinib mesylate, nilotinib, and dasatinib. Dermatol Ther..

[42] Druker BJ, Talpaz M, Resta DJ (2001). Efficacy and safety of a specific inhibitor of the BCR-ABL tyrosine kinase in chronic myeloid leukemia. N Engl J Med..

[43] Valeyrie L, Bastuji-Garin S, Revuz J (2003). Adverse cutaneous reactions to imatinib (STI571) in Philadelphia chromosome-positive leukemias: a prospective study of 54 patients. J Am Acad Dermatol..

[44] Milojkovic D, Short K, Salisbury JR (2003). Dose-limiting dermatological toxicity secondary to imatinib mesylate (STI571) in chronic myeloid leukaemia. Leukemia.

[45] Hsiao LT, Chung HM, Lin JT (2002). Stevens-Johnson syndrome after treatment with STI571: a case report. Br J Haematol.

[46] Tsao AS, Kantarjian H, Cortes J (2003). Imatinib mesylate causes hypopigmentation in the skin. Cancer.

[47] Arora B, Kumar L, Sharma A (2004). Pigmentary changes in chronic myeloid leukemia patients treated with imatinib mesylate. Ann Oncol.

[48] Aleem A (2009). Hypopigmentation of the skin due to imatinib mesylate in patients with chronic myeloid leukemia. Hematol Oncol Stem Cell Ther..

[49] Li CC, Malik SM, Blaeser BF, Dehni WJ, Kabani SP, Boyle N (2012). Mucosal pigmentation caused by imatinib: report of three cases.. Head Neck Pathol..

[50] Sidoroff A, Halevy S, Bavinck JN (2001). Acute generalized exanthematous pustulosis (AGEP)-a clinical reaction pattern.. J Cutan Pathol..

[51] Liu D, Seiter K, Mathews T (2004). Sweet’s syndrome with CML cell infiltration of the skin in a patient with chronic-phase CML while taking Imatinib Mesylate. Leuk Res.

[52] Pérez NO, Esturo SV, Viladomiu Edel A (2014). Pseudoporphyria induced by imatinib mesylate. Int J Dermatol.

[53] Rousselot P, Larghero J, Raffoux E (2003). Photosensitization in chronic myelogenous leukaemia patients treated with imatinib mesylate. Br J Haematol.

[54] Woo SM, Huh CH, Park KC, Youn SW (2007). Exacerbation of psoriasis in a chronic myelogenous leukemia patient treated with imatinib. J Dermatol.

[55] Drummond A, Micallef-Eynaud P, Douglas WS (2003). A spectrum of skin reactions caused by the tyrosine kinase inhibitor imatinib mesylate (STI 571, Glivec). Br J Haematol..

[56] Kantarjian H, Giles F, Wunderle L (2006). Nilotinib in imatinib-resistant CML and Philadelphia chromosome-positive ALL. N Engl J Med..

[57] Cortes JE, Kim DW, Pinilla-Ibarz J (2013). A phase 2 trial of ponatinib in Philadelphia chromosome-positive leukemias.. N Engl J Med..

[58] Cortes JE, Kantarjian HM, Brümmendorf TH (2011). Safety and efficacy of bosutinib (SKI-606) in chronic phase Philadelphia chromosome-positive chronic myeloid leukemia patients with resistance or intolerance to imatinib. Blood.

[59] McLellan B, Kerr H (2011). Cutaneous toxicities of the multikinase inhibitors. Dermatol Ther..

[60] Lipworth AD, Robert C, Zhu AX (2009). Hand-foot syndrome (handfoot skin reaction, palmar-plantar erythrodysesthesia): focus on sorafenib and sunitinib.. Oncology.

[61] Chu EY, Wanat KA, Miller CJ, Amaravadi RK, Fecher LA, Brose MS (2012). Diverse cutaneous side effects associated with BRAF inhibitor therapy: A clinicopathologic study. J Am Acad Dermatol.

[62] Lee WL, Lee JL, Chang SE, Lee MW, Kang YK, Choi JH (2009). Cutaneous adverse effects in patients treated with the multitargeted kinase inhibitors sorafenib and sunitinib.. Br J Dermatol.

[63] McLellan B, Kerr H (2011). Cutaneous toxicities of the multikinase inhibitors sorafenib and sunitinib. Dermatol Ther.

[64] Autier J, Escudier B , Wechsler J, Spatz A, Robert C (2008). Prospective study of the cutaneous adverse effects of sorafenib, a novel multikinase inhibitor. Arch Dermatol.

[65] Jiménez-Gallo D, Albarrán-Planelles C, Linares-Barrios M (2013). Eruptive melanocytic nevi in a patient undergoing treatment with sunitinib. JAMA Dermatol.

[66] Ikeda M, Fujita T, Mii S (2012). Erythema multiforme induced by sorafenib for metastatic renal cell carcinoma. Jpn J Clin Oncol.

[67] Suwattee P, Chow S, Berg BC, Warshaw EM (2008). Sunitinib: a cause of bullous palmoplantar erythrodysesthesia, periungual erythema, and mucositis. Arch Dermatol.

[68] Robert C, Mateus C, Spatz A (2009). Dermatologic symptoms associated with the multikinase inhibitor sorafenib. J Am Acad Dermatol.

[69] Nadauld LD, Miller MB, Srinivas S (2011). Pyoderma gangrenosum with the use of sunitinib. J Clin Oncol..

[70] Chappell JA, Burkemper NM, Semchyshyn N (2009). Localized dyskeratotic plaque with milia associated with sorafenib. J Drugs Dermatol..

[71] Dubauskas Z, Kunishige J, Prieto VG (2009). Cutaneous squamous cell carcinoma and inflammation of actinic keratoses associated with sorafenib. Clin Genitourin Cancer.

[72] Kong HH, Cowen EW, Azad NS (2007). Keratoacanthomas associated with sorafenib therapy. J Am Acad Dermatol..

[73] Jantzem H, Dupre-Goetghebeur D, Spindler P, Merrer J (2009). Sorafenib-induced multiple eruptive keratoacanthomas. Ann Dermatol Venereol.

[74] Baldo BA (2013). Adverse events to monoclonal antibodies used for cancer therapy. Oncoimmunology.

[75] Weber J (2007). Review: anti-CTLA-4 antibody ipilimumab: case studies of clinical response and immune-related adverse events. Oncologist.

[76] Kahler KC, Hauschild A (2011). Treatment and side effect management of CTLA-4 antibody therapy in metastatic melanoma. J Dtsch Dermatol Ges.

[77] Trefzer U, Minor D, Ribas A, Lebbe C, Siegfried A, Arya N (2011). BREAK-2: a phase IIA trial of the selective BRAF kinase inhibitor GSK2118436 in patients with BRAF mutation-positive (V600E ⁄ K) metastatic melanoma. Pigment Cell Res.

[78] Sheik Ali S, Goddard AL, Luke JJ (2015). Drug-associated dermatomyositis following ipilimumab therapy: a novel immune-mediated adverse event associated with cytotoxic T-lymphocyte antigen 4 blockade. JAMA Dermatol.

[79] Di Giacomo AM, Biagioli M, Maio M (2010). The emerging toxicity profiles of anti-CTLA-4 antibodies across clinical indications. Semin Oncol.

[80] Manousaridis I, Mavridou S, Goerdt S, Leverkus M, Utikal J (2013). Cutaneous side effects of inhibitors of the RAS/RAF/MEK/ERK signalling pathway and their management. J Eur Acad Dermatol Venereol.

[81] Anforth R, Fernandez-Penas P, Long GV (2013). Cutaneous toxicities of RAF inhibitors. Lancet Oncol..

[82] Flaherty KT, Puzanov I, Kim KB, Ribas A, McArthur GA, Sosman JA (2010). Inhibition of mutated, activated BRAF in metastatic melanoma. N Engl J Med.

